# The value of age and medical history for predicting colorectal cancer and adenomas in people referred for colonoscopy

**DOI:** 10.1186/1471-230X-11-97

**Published:** 2011-09-08

**Authors:** Barbara-Ann Adelstein, Petra Macaskill, Robin M Turner, Peter H Katelaris, Les Irwig

**Affiliations:** 1 Prince of Wales Clinical School, Faculty of Medicine, University of New South Wales, Sydney, Australia; 2 Screening and Test Evaluation Program, Sydney School of Public Health, University of Sydney University, NSW, Australia; 3 Gastroenterology Department, Concord Hospital, University of Sydney, NSW, Australia

## Abstract

**Background:**

Colonoscopy is an invasive and costly procedure with a risk of serious complications. It would therefore be useful to prioritise colonoscopies by identifying people at higher risk of either cancer or premalignant adenomas. The aim of this study is to assess a model that identifies people with colorectal cancer, advanced, large and small adenomas.

**Methods:**

Patients seen by gastroenterologists and colorectal surgeons between April 2004 and December 2006 completed a validated, structured self-administered questionnaire prior to colonoscopy. Information was collected on symptoms, demographics and medical history. Multinomial logistic regression was used to simultaneously assess factors associated with findings on colonoscopy of cancer, advanced adenomas and adenomas sized 6 -9 mm, and ≤ 5 mm. The area under the curve of ROC curve was used to assess the incremental gain of adding demographic variables, medical history and symptoms (in that order) to a base model that included only age.

**Results:**

Sociodemographic variables, medical history and symptoms (from 8,204 patients) jointly provide good discrimination between colorectal cancer and no abnormality (AUC 0.83), but discriminate less well between adenomas and no abnormality (AUC advanced adenoma 0.70; other adenomas 0.67). Age is the dominant risk factor for cancer and adenomas of all sizes. Having a colonoscopy within the last 10 years confers protection for cancers and advanced adenomas.

**Conclusions:**

Our models provide guidance about which factors can assist in identifying people at higher risk of disease using easily elicited information. This would allow colonoscopy to be prioritised for those for whom it would be of most benefit.

## Background

The majority of colorectal cancer cases are still diagnosed in a clinical setting, even in countries offering screening [1]. Early detection of colorectal cancer reduces both the cancer mortality [2-7] and the incidence [6,8] of the disease. The reduction in incidence is thought to be due to removal at colonoscopy of adenomas which are recognised as non-obligate precursor lesions to cancer [9]. Adenomas are commonly found in adults over 50 years undergoing colonoscopy, and the majority of these will not develop into cancer. The malignant potential of an adenoma depends on its size and histology, with larger adenomas and those with more than 25% villous architecture or dysplasia more likely to have a higher risk. Church has estimated that 16% of adenomas between 6-9 mm and 4% of those between 1-5 mm fit into this higher risk category based on their histology [10].

Colonoscopy is an invasive and costly procedure: serious complications, including bleeding and bowel perforation may occur in 0.1-0.6% of procedures [11-13]. Further, colonoscopy is a scarce resource. The Center for Disease Control and Prevention (CDC) in the United States has shown that even if half the colonoscopy capacity was dedicated to screening, the capacity to undertake such screening would be limited [14]. It would therefore be useful to be able to select people for colonoscopy who at higher risk of either cancer or premalignant adenomas.

We have previously shown that symptoms are not good predictors of the presence of colorectal cancer but that prediction improved using a model which also included demographic details and medical history such as previous colonoscopy, bowel disease, smoking history, and use of aspirin or non steroidal anti-inflammatory medications [15]. Advanced adenomas were excluded from that analysis. The aim of this current paper is to report the results of a more comprehensive model that discriminates between colorectal cancer, advanced adenoma, large adenomas and small adenomas (versus none of these abnormalities).

## Methods

The study design and inclusion criteria have been described previously [15]. This is a cross sectional study in which participating patients (> 18 years, and recruited from participating gastroenterologists and colorectal surgeons following scheduling for colonoscopy for any indication) completed a questionnaire, previously shown to be reliable and reproducible [16], eliciting details about demographic details, family history, medical history (previous colonoscopy and bowel disease, polyps, aspirin and NSAID use within the previous 2 years, smoking history), and bowel symptoms. These included rectal bleeding, change in bowel habit, passage of rectal mucus, abdominal or anal pain, sensation of abdominal or anal lump, incomplete evacuation, urgency, history of anaemia, weight loss, and fatigue. We also elicited information about characteristics of the symptom such as duration, frequency and severity and whether or not the patients had sought consultation with a doctor for that symptom.

Findings at colonoscopy were obtained from endoscopic records. All lesions found were confirmed by histological examination. We classified findings into five categories: cancer, advanced adenoma (adenoma with significant (> 25%) villous features, or high grade dysplasia, including carcinoma-in-situ, or size 10 mm or larger [17,18], adenomas 6 to 9 mm in size, adenomas ≤ 5 mm, and no adenoma found. Where patients had more than one lesion, we classified them by their most significant lesion, according to the hierarchy listed above. If the adenoma size was not recorded by the endoscopist, we used the size recorded at histological examination; if neither of these was available, we used the size description noted by the endoscopist (based on analysis of adenomas for which we had both the description and size reported, we categorised descriptions of "diminutive", "tiny", "very small", or "minor" as ≤ 5 mm; "moderate" or "small" as 6-9 mm; and "large", "very large", or "huge" as ≥ 10 mm). We classified adenomas for which no size was recorded as ≤ 5 mm (n = 32).

Patients were recruited between April 2004 and December 2006. We included only patients who completed the questionnaire 6 months or less before their colonoscopy was done, and whose colon examination was complete (visualisation of the caecal pole at colonoscopy or if not visualised, by follow up bowel investigations).

### Ethics committee approval

The study received approval from the Ethics Committees of the University of Sydney, Central Sydney (CRGH and Central Zones), Northern Sydney and Central Coast, and Western Sydney Area Health Services and the Sydney Adventist Hospital. All patients provided written consent.

### Statistical Analysis

Patients were grouped according to the most significant grade of abnormality found giving five different outcome groups: cancer, advanced adenomas, adenomas of size 6 to 9 mm, adenomas of size ≤ 5 mm, and no cancer or any adenomas. Descriptive analyses were undertaken to assess the prevalence of cancer, advanced adenomas and smaller adenomas separately by demographic, medical history and symptom variables using the sum of no abnormality and the outcome of interest only as the denominator. Odds ratios were calculated comparing the odds of having cancer, advanced adenoma or adenomas 6-9 mm or ≤ 5 mm (separately for each of these outcomes) with no abnormality univariately for each of the symptom, demographic and other health information subgroups. Multinomial logistic regression was then used to simultaneously assess which of these risk factors were associated with the outcomes of cancer, advanced adenomas and adenomas sized 6 - 9 mm, and ≤ 5 mm. This method fits simultaneous logistic regression models, each with its own intercept and coefficients, to compare each of the four outcomes listed above to the referent category (no cancer, advanced adenoma or adenoma of any size). Backwards elimination of risk factors was used to simplify the model using likelihood ratio tests with p < 0·05 as the criterion for statistical significance. Interactions were considered for elimination first. As numerous comparisons were made, results for interactions were not included if their significance was close to 0·05 and there was no biologically plausible basis for the interaction. Because the estimated coefficients for the explanatory variables vary by outcome, odds ratios for the final model were calculated for each risk factor (compared to not having the risk factor) for each outcome of cancer, advanced adenoma and adenoma of any size using the absence of these as the reference group. This analysis for the cancer outcome differs slightly from that reported previously [15] as the comparison group in that paper included adenomas less than 10 mm in the referent group, whereas this analysis uses no abnormality as the referent group.

A sequence of additional multinomial logistic regression models were fitted to assess the incremental value of variables found to be statistically significant in the final model. The sequence was: (1) age only; (2) model 1 + other demographic variables; (3) model 2 + medical history variables; (4, the final model) model 3 + symptoms. For each of the four outcomes, estimates of sensitivity and specificity across all values of predicted probability, with no abnormality (ie no cancer, advanced adenoma or adenomas) as the referent group common to all, were used to obtain a ROC curve. The area under each curve was used to assess the ability of the model to discriminate between patients with no abnormality and patients with (i) adenomas ≤ 5 mm; (ii) adenomas 6 - 9 mm; (iii) advanced adenomas and (iv) cancer.

The percent of abnormalities that would have been detected was calculated for different possible screening criteria based on age and previous colonoscopy. To allow comparison with the model, the predicted probabilities of cancer were sorted from highest to lowest and a cut-point was applied to include the same number of patients (above the cut-point) who would have been screened based on the age and previous colonoscopy criteria. Detection rates for cancer and adenomas were then compared. Detection rates for the 40% of patients with the highest predicted probability of cancer from the model were also computed. All analyses were done in SAS version 9.2.

## Results

**Figure 1:**
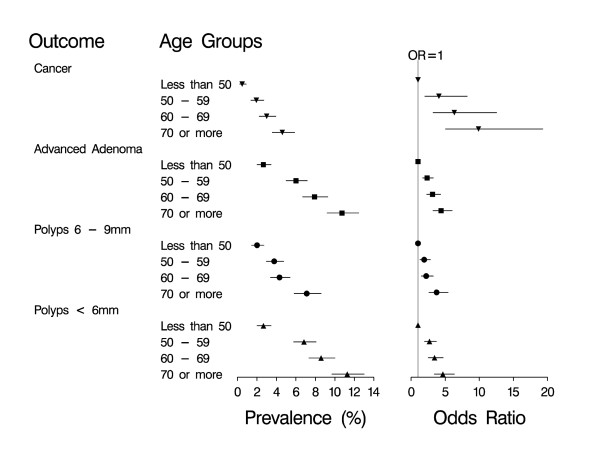
**Colorectal cancer and adenomas: prevalence and risk of different age groups**. The prevalence (with 95% confidence interval) of cancer, advanced adenomas and adenomas (≤5 mm and 6 - 9 mm) for each age group (less than 50 years, 50 - 59 years, 60 - 69 years and more than 70 years) and the odds ratio (with 95% confidence interval) of having cancer, advanced adenomas and adenomas for the age groups 50 - 59 years, 60 - 69 years and more than 70 years compared to those less than 50 years. 6784 patients had no cancer, advanced adenomas or adenomas, 507 had adenomas ≤ 5 mm, 286 had adenomas 6 - 9 mm, 468 had advanced adenomas and 159 had cancer.

Data were available from a total of 8,204 patients. 47% were male. The age range was 18 to 95 years (median age 58 years), with 27% aged less than 50 years, 26% 50-59 years, 25% 60-69 years and 22% over 70 years of age. All patients underwent colonoscopy, for which there was a 98% caecal intubation rate. The overall cancer prevalence was 1.9% (159 patients). Risk of cancer and adenomas was dependent on age (Figure 1). The prevalence of cancer and all types of adenomas ranged from less than 3% in people under 50 years of age, and increased to over 10% in people 70 years or older for advanced adenoma. The odds ratios, which measure the increase in prevalence as age increases relative to people under 50 was strongest for cancers, and similar for all types of adenomas.

59% had undergone colonoscopy in the previous 10 years. Cancer rates were approximately 5 times greater in patients who had not had a previous colonoscopy in each age group, whereas advanced adenoma rates were about twice as high in people who had not had a colonoscopy in each age group (Table 1). For smaller adenomas (less than 10 mm), there was no clear pattern related to prior colonoscopy.

**Table 1:** Rates (per 1,000) of cancers and adenomas by age and colonoscopy in previous 10 years

	Cancer n = 159		Advanced Adenoma n = 468		Adenoma 6 - 10 mm n = 286		Adenoma ≤ 5 mm n = 507		No abnormality n = 6784	
Age Group (years)	**Cpy** **yes**	**Cpy** **no**	**Cpy** **yes**	**Cpy** **no**	**Cpy** **yes**	**Cpy** **no**	**Cpy** **yes**	**Cpy** **no**	**Cpy** **yes**	**Cpy** **no**

≤50	1	6	19	29	20	18	38	19	922	928
50 - 59	4	31	35	74	33	33	60	63	867	800
60 - 69	11	48	54	93	34	39	84	60	817	760
≥70	17	74	78	109	57	53	94	90	754	674

Total	9	31	50	66	38	31	73	49	831	822

Cpy = colonoscopy in previous 10 years

The relative prevalence of abnormalities increased with age in a similar way for those who had or had not had a previous colonoscopy. For instance, in people who have had a previous colonoscopy, the cancer rate in people aged 70 or more was 17 times higher than in people aged less than 50 (from Table 1, 17/1000 divided by 1/1000). The estimate in people who have not had a previous colonoscopy in the previous 10 years was 12.3 (from Table 1, 74/1000 divided by 6/1000). The corresponding estimates for: advanced adenoma were 4.1 vs 3.8; adenomas 6-10 mm were 2.9 vs 2.9; adenomas ≤ 5 mm were 2.5 vs 4.7 respectively. There is no statistical evidence (p = 0.51) that the effect of age was modified by previous colonoscopy which indicates that the observed variability is due to chance. Furthermore, the predicted rates per 1000 based on a multinomial model (see additional file 1) that included age and previous colonoscopy as predictor variables are very similar to the observed rates in Table 1, indicating that even with only these two variables the model fits well.

The fact that the model with the 2 major predictors, age and previous colonoscopy, fits the data well suggests that the appropriate way to assess the value of potential risk factors is to assess whether adding them to this multivariable model results in any improvement to the fit. Further model checks showed that the odds ratios for all predictors were very similar in the model that included all additional significant variables, when run separately for people with or without previous colonoscopy (additional file 1). Similarly, statistical tests for interaction indicated that neither previous colonoscopy nor age were effect modifiers for the other variables subsequently included in the model.

### Multivariable risk identification

The results of the multivariable model to distinguish between each outcome and the absence of cancer or any adenoma (the referent category) are shown in Table 2. The effect of age followed a similar pattern to that in Figure 1. For cancer and all types of adenomas, male gender increased the risk by about a third. Colonoscopy in the last 10 years remained highly protective for cancer, but the effect became far less marked the smaller the adenoma. With previous colonoscopy in the model, a history of adenomas was not predictive of cancer, but was predictive of finding adenomas again. There was an exposure response relationship between amount of tobacco smoked and cancer. For adenomas, the gradient of the exposure-response was less steep, and became flatter the smaller the size of the adenoma. A self reported history of irritable bowel syndrome, and of NSAID or aspirin use was associated with reduced cancer risk but the effect on adenomas was weaker or absent. For symptoms, a history of passing mucus per rectum and rectal bleeding were associated with a higher risk of cancer, particularly if the symptom was of recent onset and occurred frequently. This association was not evident for people with adenomas, with the possible exception of bleeding with advanced adenomas.

**Table 2:** Multinomial model odds ratios for the included demographic/medical history and symptom variables for each of the four outcomes compared to patients with no adenomas, advanced adenomas or cancers

				Cancer (n = 159)	Advanced Adenoma (n = 468)	Adenoma Size 6 - 9 mm (n = 286)	Adenoma Size ≤ 5 mm (n = 507)	
		**n**	**%**	**OR (95% CI)**	**OR (95% CI)**	**OR (95% CI)**	**OR (95% CI)**	**p-value**
DEMOGRAPHIC/MEDICAL HISTORY								
Age	< 50 years (Reference)	2211	27.0					
	50 - 59	2169	26.4	6.84 (3.33, 14.06) **	2.54 (1.82, 3.55) **	1.72 (1.15, 2.55)**	2.36 (1.70, 3.26)**	<.001
	60 - 69	2032	24.8	13.84 (6.77, 28.29) **	3.47 (2.48, 4.87) **	1.79 (1.19, 2.70) **	2.74 (1.97, 3.82)**	
	70 or more	1792	21.8	23.54 (11.43, 48.45) **	5.27 (3.72, 7.47) **	2.99 (1.98, 4.52) **	3.60 (2.55, 5.07) **	
Gender	Female (Reference)	4344	52.9					
	Male	3860	47.1	1.44 (1.02, 2.04)*	1.29 (1.05, 1.57)*	1.53 (1.19, 1.96)**	1.37 (1.13, 1.65)**	<.001
Colonoscopy	None in last 10 years (Reference)	3791	46.2					
	Previous (in last 10 years)	4413	53.8	0.22 (0.15, 0.34) **	0.42 (0.33, 0.53) **	0.71 (0.53, 0.95)*	0.87 (0.70, 1.09)	<.001
History of colorectal polyps	No (reference)	6436	78.4					
	Yes	1768	21.6	0.87 (0.47, 1.59)	1.99 (1.53, 2.58) **	1.89 (1.39, 2.56) **	1.79 (1.43, 2.25) **	<.001
Smoking Status	Non-smoker (Reference)	4710	57.4					
	4 or less pack years	1246	15.2	0.89 (0.52, 1.55)	0.95 (0.70, 1.30)	0.75 (0.51, 1.12)	0.89 (0.67, 1.19)	0.010
	4 to 15.5 pack years	1188	14.5	1.49 (0.95, 2.33)	1.43 (1.09, 1.86) **	1.04 (0.74, 1.48)	0.95 (0.73, 1.25)	
	more than 15.5 pack years	1060	12.9	1.52 (0.98, 2.37)	1.53 (1.17, 2.00) **	1.40 (1.01, 1.93)*	1.11 (0.85, 1.45)	
History of irritable bowel syndrome	No (Reference)	7240	88.2					
	Yes	964	11.8	0.45 (0.21, 0.99)*	0.45 (0.29, 0.70) **	0.98 (0.66, 1.45)	0.95 (0.71, 1.29)	0.001
History of NSAID use	No (Reference)	7445	90.7					
	Yes	759	9.3	0.33 (0.15, 0.72) **	0.42 (0.28, 0.65) **	0.80 (0.52, 1.22)	0.69 (0.49, 0.97)*	<.001
History of aspirin use	No (Reference)	6874	83.8					
	Yes	1330	16.2	0.54 (0.34, 0.85) **	0.79 (0.61, 1.02)	1.09 (0.81, 1.48)	0.94 (0.74, 1.20)	0.023
Education Level	Secondary or lower (Reference)	3721	45.4					
	Tertiary	4483	54.6	0.83 (0.59, 1.17)	0.81 (0.67, 0.99)*	1.03 (0.81, 1.33)	0.80 (0.66, 0.97)*	0.044
SYMPTOMS								
Bleeding	No Symptom (Reference)	5181	63.2					
	no other info	64	0.8	2.35 (0.67, 8.18)	0.51 (0.12, 2.16)	1.52 (0.53, 4.31)	0.22 (0.03, 1.60)	<.001
	present greater 12 months	1044	12.7	1.25 (0.68, 2.33)	1.39 (1.03, 1.88)*	0.67 (0.42, 1.05)	1.04 (0.77, 1.40)	
	Occurring monthly or occasionally; and present less 12 months	1226	14.9	2.00 (1.26, 3.17) **	1.21 (0.92, 1.60)	1.08 (0.77, 1.52)	1.08 (0.83, 1.42)	
	weekly; present less 12 months	689	8.4	5.09 (3.26, 7.94) **	1.43 (1.00, 2.03)	0.80 (0.47, 1.36)	0.58 (0.36, 0.93)*	
Mucus	No Symptom (Reference)	6886	83.9					
	no other info	131	1.6	0.43 (0.06, 3.24)	1.01 (0.43, 2.37)	0.27 (0.04, 1.96)	0.55 (0.20, 1.51)	0.001
	present greater 12 months	466	5.7	1.10 (0.43, 2.79)	0.69 (0.39, 1.20)	0.69 (0.35, 1.37)	0.60 (0.36, 1.02)	
	Occurring monthly or occasionally; and present less 12 months	393	4.8	1.37 (0.66, 2.84)	0.82 (0.48, 1.42)	1.71 (1.04, 2.82)*	0.99 (0.62, 1.60)	
	Weekly; present less 12 months	328	4.0	3.00 (1.71, 5.25) **	0.99 (0.57, 1.72)	0.46 (0.17, 1.26)	0.35 (0.14, 0.85)*	
Anaemia	No (Reference)	7383	90.0					
	Yes	821	10.0	2.95 (1.96, 4.45) **	0.84 (0.59, 1.21)	0.55 (0.32, 0.94)*	0.76 (0.54, 1.09)	<.001
Fatigue	No (Reference)	4938	60.2					
	Yes	3266	39.8	1.36 (0.97, 1.91)	0.67 (0.54, 0.83) **	0.80 (0.62, 1.04)	0.90 (0.74, 1.10)	<.001

Total number of patients 8204; number with no abnormality 6784.

Key ORs in bold are statistically significant

* p 0.01 - 0.05

** p < 0.01

**Figure 2:**
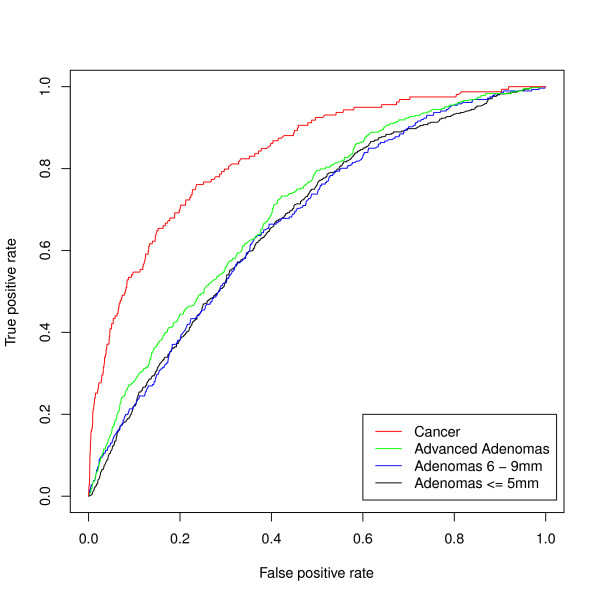
**ROC curves for the multinomial model showing the discrimination of the model for cancer, advanced adenomas and adenomas sized 5 - 9 mm and ≤ 5 mm; for each outcome the reference group is no cancer, advanced adenoma, or adenomas**.

The areas under the ROC curves based on the predictive models for cancer and adenomas are shown in Figure 2 and Table 3. With only age in the model, the areas were 0.66 for cancer and between 0.60 and 0.62 for adenomas indicating moderate discrimination. As other sociodemographic variables, medical history and symptoms were added, the area under the curve for cancer achieved good discrimination (0.83), but the improvement for advanced adenomas and smaller adenomas was less marked (0.70 and 0.67 respectively).

**Table 3:** Areas under the curve for multivariable prediction of cancer and adenomas

	Cancer	Advanced Adenoma	Adenomas 6 - 9 mm	Adenomas ≤5 mm
*Model 1*: Age	0.66	0.62	0.60	0.62
*Model 2*: Model 1 + gender, previous colonoscopy, education level	0.77	0.66	0.62	0.64
*Model 3*: Model 2 + history of: smoking, IBS, adenomas, or NSAID, aspirin use	0.79	0.69	0.65	0.66
*Model 4*: Model 3 + symptoms*	0.83	0.70	0.67	0.67

Note: NSAID = non- steroidal anti-inflammatory drug

IBS = irritable bowel syndrome

*only bleeding, rectal mucus, anaemia and fatigue were found to be significant and included in the final model.

Using the multivariable model (which includes age, sociodemographic variables and symptoms), the predicted probability for an individual can be calculated for each of the outcomes, based on age and gender, medical history and symptoms. The value of the model can be demonstrated by comparing the model results with simple methods for predicting risk (Table 4). For example, if we restricted colonoscopies to people 40 years and older, we would have avoided 10.5% of colonoscopies but still detected all of the cancers and over 97% of the adenomas. If we use the model to avoid colonoscopy in the 10.5% at lowest cancer risk, we would have missed 1.3% of the cancers. So for low-risk patients, a simple age-based method does well. For high-risk patients defined for example as people 60 years and older who have not had a colonoscopy in the past 10 years: 16% of the population are in this group but it contains almost half of the cancers (49.1%) and 28% of the advanced adenomas. If we examine instead the 16% at highest risk from the model, the cancer detection rate increases to 64.8%, without any loss in adenoma detection. In fact 85.5% of the cancers and 57.7% of the adenomas could be detected by the model by only performing colonoscopy on the 40% identified by the model as being at highest risk.

**Table 4:** Outcome (percentage) that would have been detected using the model if colonoscopy restricted

Group to which colonoscopy restricted	Cancer (%)	Advanced Adenoma (%)	Adenomas 6 - 9 mm (%)	Adenomas ≤ 5 mm (%)
Aged 40+ (89.5% of patients)	100.0	97.0	97.2	97.4
Top 89.5% predicted probability of cancer from model	98.7	96.8	93.0	92.5
Aged 60+ and no previous colonoscopy (16% of patients)	49.1	28.2	20.6	18.9
Top 16% predicted probability of cancer from model	64.8	28.2	16.8	17.8

## Discussion

Our findings demonstrate that a predictive model based on sociodemographic variables (age, gender and education level), pertinent medical history (previous colonoscopy, smoking, use of NSAID or aspirin, previous polyps, and IBS) and symptoms (rectal bleeding, rectal mucus, anaemia and fatigue), does well at predicting colorectal cancer and reasonably well at predicting advanced adenomas.

It is of interest to identify which variables are most strongly predictive of cancer and adenoma prevalence. Age is the dominant risk factor for cancer and for adenomas of all sizes. Having had a colonoscopy within the previous 10 years confers protection for cancers and advanced adenomas. Adding medical history and symptoms (rectal bleeding, mucus, anaemia and fatigue) to the model adds further modest improvement to cancer prediction, but negligible improvement to adenoma prediction.

Our finding that family history is not associated with an increase in prevalence of colorectal cancer may seem surprising. It is likely that this reflects the clinical setting of our cohort, with patients with a family history of colorectal cancer already having been screened and included in those having undergone colonoscopy previously. Other studies have also noted that in people with symptoms a positive family history does not increase the cancer prevalence [19,20], and indeed, guidelines for referral of patients in place in Britain which aim to identify patients with higher risk symptoms, do not include assessment of family history [21].

The quality of our study relates to several factors including the size of our study with over 8,000 patients, the prospective nature of the data collection, the completeness of information on all patients, the requirements of complete examination of the entire colon, and pathological examination of all lesions encountered. Information about symptoms was also consistently collected using a validated questionnaire [16]. A further strength of our study is that it represents a heterogeneous population which reflects what occurs in clinical practice in the real world and allows exploration of what factors that make up that heterogeneity predict the probability of cancer or adenomas. A potential limitation of our study was that there was no standard reporting for colonoscopy. However, the reports from which data were extracted were those used in clinical practice; based on a caecal intubation rate of 98% we believe the procedures were of high quality.

Our model does well at predicting cancer prevalence, achieving an area under the ROC curve of 0.83 which is similar to that found in other studies, for example Selvachandran (0.86) [22]. Our models help to identify individuals who have a high probability of cancer amongst people referred to gastroenterologists and colorectal surgeons, thus helping to indicate the urgency for colonoscopy. At the low-risk end of the spectrum, prediction can be simplified to age: the probability of cancer or adenoma is very low in people under 40 and reduced still further if they have had a colonoscopy in the previous 10 years. For them, potential risks of colonoscopy may outweigh potential benefits. Consideration can be given to discussing benefits and harms of the procedure with patients to reach the best benefit-harm trade-off for each person, as has been done in other areas of health care [23].

In addition, risk information from the model can be useful at a policy level. Decision making about resource utilisation at a population level should take risk assessment into account to ensure that colonoscopy is prioritised to groups at higher risk of disease. At a general practice level, resources may, for example be directed to ensure that those in higher risk groups are referred for colonoscopy, while at a specialist level resources should be targeted at those who have never had a colonoscopy rather than for inappropriate, frequent colonoscopy. At a population level, symptoms as warnings for cancer or adenomas should be de-emphasised. Our model is not strictly applicable to patients presenting to a general practice. However, it is not feasible to do a study in patients presenting to a general practitioner and obtain colonoscopies on all patients. Indeed, the major symptom prediction studies in patients have been done in referred populations [22,24,25]. Our cancer prevalence is considerably lower (1.9%) than in other similar studies, which report cancer prevalences of between 4 to 12% [22,24-26], suggesting that our population is less strongly filtered and therefore more representative of general practice.

In addition, given that in general practice the probability of cancer may be even lower than that predicted in the referred population, it seems reasonable to use the information from the model to inform decisions in general practice, in particular to identify who has a very low probability of cancer or advanced adenoma. The model will be the most reliable source of predicting cancer or advanced adenoma for most patient characteristics. This can be supplemented with selected information, for example the effect of family history, from sources where that has been reliably estimated elsewhere.

Another approach to identifying patients at higher risk for cancer or adenomas on colonoscopy patients is to use FOBTs [2-5], as suggested by Rozen [27]. A recent review of FOBTs provided odds ratios for FOBT detection of cancer and advanced adenoma, which can be converted to areas under the ROC curve (AUC) and compared with our model [28]. The AUC values were 0.93 for cancer, 0.88 for advanced adenomas and 0.69 for all adenomas. Other AUC values obtained for adenomas in a clinically presenting population were 0.72 for advanced adenomas and 0.64 for all adenomas. Overall, these are similar to or slightly higher than those found in our study. These data might suggest that FOBT would be as, or more, effective than our model as a triage tool for prioritising colonoscopy. However, FOBT requires additional cost and effort, whereas our model requires only easily and immediately obtainable sociodemographic and medical history information. Models that incorporate both this information and FOBT results should be developed and evaluated as this may boost prediction still further.

## Conclusions

Colorectal cancer is common and preventable. Our models may assist in identifying population subgroups at higher risk of disease, ensuring that colonoscopy is prioritised for those for whom it would be of most benefit. Age is the dominant risk factor in this model. Younger age and prior colonoscopy in the preceding 10 years predicts a low probability of cancer or adenomas and should be appreciated by referrers, proceduralists, providers and health planners when aiming to target colonoscopy resources most effectively.

## Competing interests

The authors declare that they have no competing interests.

## Authors' contributions

BA, LI and PM conceived the study; BA implemented the study and collected the data; RT and PM did the statistical analysis; BA, LI, RT and PM wrote the manuscript, although all authors contributed. All authors contributed to the interpretation of the data and were responsible for reviewing the manuscript. All authors have read and approved the final manuscript.

## Authors' information

Barbara-Ann Adelstein, senior research fellow, Prince of Wales Clinical School, Faculty of Medicine, University of NSW, Sydney, Australia.

Petra Macaskill, associate professor, STEP, The Sydney School of Public Health, University of Sydney, Australia.

Robin M Turner, research fellow biostatistics, STEP, The Sydney School of Public Health, University of Sydney, Australia.

Peter H Katelaris, gastroenterologist, Department of Gastroenterology, Concord Hospital, University of Sydney, Australia.

Les Irwig, professor of epidemiology, STEP, The Sydney School of Public Health, University of Sydney, Australia.

## Pre-publication history

The pre-publication history for this paper can be accessed here:

http://www.biomedcentral.com/1471-230X/11/97/prepub

## Supplementary Material

Additional file 1 **Table 1: Multinomial model results from two separate models for 1) those patients who have had a colonoscopy in the last 10 years, and 2) those patients who have not**. Table showing odds ratios for each variable in the multinomial model for cancer, advanced adenoma, adenomas 6-9 mm, and adenomas ≤ 5 mm, shown for patients who have had a colonoscopy in the last 10 years and for those who have not. Click here for file
