# Supplementary material for: The value of age and medical history for predicting colorectal cancer and adenomas in people referred for colonoscopy
Source: BMC Gastroenterol. 2011 Sep 8;11:97. doi: 10.1186/1471-230X-11-97 (PMC3175197; doi:10.1186/1471-230X-11-97)
Supplement: Additional file 1 — Table 1: Multinomial model results from two separate models for 1) those patients who have had a colonoscopy in the last 10 years, and 2) those patients who have not. Table showing odds ratios for each variable in the multinomial model for cancer, advanced adenoma, adenomas 6-9 mm, and adenomas ≤ 5 mm, shown for patients who have had a colonoscopy in the last 10 years and for those who have not. [file 1471-230X-11-97-S1.PDF]

Additional File:

Table 1: Multinomial model results from two separate models for 1) those patients who have had a colonoscopy in the last 10 years, and 2) those patients who have not.

	Cancer		Advanced Adenoma		Adenoma 6-9 mm		Adenoma ≤5mm	
	Previous Cppy	No Previous Cppy	Previous Cppy	No Previous Cppy	Previous Cppy	No Previous Cppy	Previous Cppy	No Previous Cppy
Variable Name	OR (95% CI)	OR (95% CI)	OR (95% CI)	OR (95% CI)	OR (95% CI)	OR (95% CI)	OR (95% CI)	OR (95% CI)
DEMOGRAPHIC / MEDICAL HISTORY								
Age (reference: < 50 years)								
50 – 59 years	7.42 (3.44, 15.99)**	5.97 (0.66, 53.61)	2.92 (1.96, 4.34)**	1.77 (0.95, 3.30)*	1.76 (1.04, 2.98)*	1.54 (0.83, 2.85)	3.62 (2.29, 5.74)**	1.41 (0.89, 2.22)
60 – 69 years	13.49 (6.21, 29.32)**	17.39 (2.14, 141.59)*	3.82 (2.51, 5.79)**	2.63 (1.44, 4.81)**	1.89 (1.08, 3.33)*	1.59 (0.86, 2.96)	3.53 (2.13, 5.83)**	1.84 (1.18, 2.88)*
70 years or more	23.64 (10.74, 52.02)**	30.66 (3.78, 248.64)**	5.47 (3.48, 8.59)**	4.06 (2.22, 7.43)**	2.88 (1.59, 5.22)	2.85 (1.55, 5.25)**	6.09 (3.62, 10.25)**	2.14 (1.36, 3.37)**
Gender (reference: Female)								
Male	1.70 (1.13, 2.56)*	0.91 (0.46, 1.79)	1.22 (0.93, 1.61)	1.36 (1.01, 1.82)*	1.36 (0.93, 2.01)	1.63 (1.16, 2.28)**	1.26 (0.92, 1.72)	1.44 (1.13, 1.84)**
Education Level (reference: secondary or lower)								
Tertiary	0.72 (0.48, 1.08)	1.20 (0.62, 2.32)	0.80 (0.60, 1.05)	0.83 (0.62, 1.11)	0.79 (0.54, 1.17)	1.26 (0.91, 1.74)	0.76 (0.55, 1.03)	0.82 (0.65, 1.05)
Smoking Status (reference: Non-smoker)								
4 or less pack years	1.02 (0.55, 1.91)	0.65 (0.19, 2.21)	0.93 (0.61, 1.43)	0.97 (0.61, 1.52)	0.58 (0.30, 1.14)	0.88 (0.53, 1.46)	1.17 (0.76, 1.79)	0.71 (0.48, 1.06)
4 to 15.5 pack years	1.57 (0.92, 2.68)	1.50 (0.66, 3.45)	1.81 (1.26, 2.59)**	1.13 (0.76, 1.67)	1.22 (0.72, 2.06)	0.93 (0.58, 1.48)	1.09 (0.69, 1.72)	0.88 (0.63, 1.24)
more than 15.5 pack years	1.69 (1.01, 2.84)*	1.30 (0.54, 3.17)	1.95 (1.34, 2.83)**	1.21 (0.82, 1.78)	1.57 (0.93, 2.63)	1.25 (0.82, 1.92)	1.28 (0.82, 2.00)	1.00 (0.72, 1.39)
History of colorectal polyps (reference: No polyps)								
Yes	1.87 (0.64, 5.45)	0.69 (0.34, 1.40)	1.09 (0.46, 2.62)	2.18 (1.64, 2.90)**	1.45 (0.51, 4.15)	1.98 (1.44, 2.73)**	0.92 (0.33, 2.61)	1.89 (1.49, 2.40)**
History of irritable bowel syndrome (reference: No)								
Yes	0.57 (0.22, 1.46)	0.30 (0.07, 1.27)	0.59 (0.30, 1.13)	0.40 (0.22, 0.71)**	1.25 (0.63, 2.47)	0.86 (0.52, 1.40)	0.68 (0.35, 1.33)	1.08 (0.77, 1.52)
History of NSAID use (reference: No)								
Yes	0.30 (0.12, 0.76)*	0.33 (0.08, 1.42)	0.44 (0.24, 0.81)**	0.42 (0.23, 0.79)**	0.71 (0.35, 1.45)	0.83 (0.49, 1.42)	0.78 (0.46, 1.35)	0.62 (0.40, 0.97)*
History of aspirin use (reference : No)								
Yes	0.43 (0.24, 0.77)**	0.80 (0.37, 1.75)	0.82 (0.57, 1.19)	0.77 (0.54, 1.11)	1.07 (0.65, 1.77)	1.11 (0.76, 1.62)	0.85 (0.56, 1.30)	1.00 (0.75, 1.34)

Table continued on next page

	Cancer		Advanced Adenoma		Adenoma 6-9 mm		Adenoma ≤5mm	
	**Previous Cppy**	**No Previous Cppy**	**Previous Cppy**	**No Previous Cppy**	**Previous Cppy**	**No Previous Cppy**	**Previous Cppy**	**No Previous Cppy**
Variable Name	**OR (95% CI)**	**OR (95% CI)**	**OR (95% CI)**	**OR (95% CI)**	**OR (95% CI)**	**OR (95% CI)**	**OR (95% CI)**	**OR (95% CI)**
**SYMPTOMS**								
Bleeding (reference : No bleeding)								
no other info	1.11 (0.14, 8.85)	4.83 (0.94, 24.76)	0.49 (0.06, 3.79)	0.50 (0.07, 3.73)	2.36 (0.66, 8.39)	0.69 (0.09, 5.21)	0.64 (0.08, 4.88)	NE
present greater 12 months	1.10 (0.52, 2.32)	1.84 (0.60, 5.60)	1.45 (0.97, 2.17)	1.32 (0.84, 2.07)	0.39 (0.18, 0.86)	0.96 (0.55, 1.69)	1.08 (0.67, 1.74)	1.06 (0.72, 1.56)
Occurs monthly/ occasionally and present less 12 months	2.03 (1.19, 3.46)	2.00 (0.78, 5.14)	1.34 (0.94, 1.93)	1.03 (0.65, 1.63)	0.77 (0.46, 1.29)	1.42 (0.90, 2.24)	1.31 (0.89, 1.93)	0.92 (0.63, 1.35)
Occurs weekly; and present less 12 months	5.52 (3.26, 9.34)**	4.33 (1.76, 10.65)**	1.73 (1.12, 2.68)*	0.95 (0.50, 1.82)	0.64 (0.29, 1.43)	0.93 (0.46, 1.90)	0.46 (0.21, 1.02)	0.67 (0.36, 1.22)
Mucus (reference: No mucus)								
no other info	0.53 (0.07, 4.27)	NE	0.63 (0.15, 2.73)	1.50 (0.52, 4.33)	NE	0.46 (0.06, 3.41)	0.87 (0.20, 3.71)	0.42 (0.10, 1.75)
present greater 12 months	1.36 (0.47, 3.99)	0.59 (0.08, 4.58)	0.53 (0.21, 1.33)	0.82 (0.41, 1.67)	0.96 (0.34, 2.73)	0.53 (0.21, 1.34)	0.65 (0.26, 1.64)	0.57 (0.30, 1.06)
Occurs monthly/ occasionally and present less 12 months	1.35 (0.58, 3.14)	1.14 (0.25, 5.23)	0.59 (0.27, 1.30)	1.16 (0.55, 2.46)	1.95 (0.97, 3.92)	1.50 (0.74, 3.08)	0.88 (0.42, 1.85)	1.12 (0.61, 2.08)
Occurs weekly; and present less 12 months	2.93 (1.49, 5.79)**	3.49 (1.23, 9.85)*	1.31 (0.69, 2.51)	0.53 (0.16, 1.73)	0.27 (0.04, 2.00)	0.60 (0.18, 1.96)	0.70 (0.25, 1.96)	0.11 (0.02, 0.80)*
Anaemia (reference: No anaemia)								
Yes	2.86 (1.74, 4.69)**	3.34 (1.56, 7.16)**	0.76 (0.46, 1.26)	0.97 (0.57, 1.65)	0.50 (0.23, 1.10)	0.56 (0.27, 1.18)	0.80 (0.48, 1.34)	0.76 (0.46, 1.24)
Fatigue (reference: No fatigue)								
Yes	1.43 (0.96, 2.12)	1.10 (0.56, 2.17)	0.61 (0.46, 0.82)**	0.76 (0.56, 1.05)	0.60 (0.40, 0.91)*	0.98 (0.69, 1.38)	1.07 (0.78, 1.46)	0.80 (0.62, 1.04)

Previous cppy = previous colonoscopy in last 10 years; No previous cppy = no previous colonoscopy in last 10 years
* p 0.01 – 0.05 **p <0.01
NE = not estimable, outcome not observed in that covariate level
